# Gene Dose-Dependent and Additive Effects of *ABCG2* rs2231142 and *SLC2A9* rs3733591 Genetic Polymorphisms on Serum Uric Acid Levels

**DOI:** 10.3390/metabo12121192

**Published:** 2022-11-29

**Authors:** Jin-Woo Park, Ji-Hyeon Noh, Jong-Min Kim, Hwa-Young Lee, Kyoung-Ah Kim, Ji-Young Park

**Affiliations:** 1 Department of Clinical Pharmacology and Toxicology, Korea University Anam Hospital, Korea University Medicine, Seoul 02841, Republic of Korea; 2 Department of Neurology, Korea University Anam Hospital, Korea University Medicine, Seoul 02841, Republic of Korea; 3 Division of Clinical Pharmacology, Department of Medicine, Vanderbilt University Medical Center, Nashville, TN 37240, USA

**Keywords:** *ABCG2*, *SLC2A9*, uric acid

## Abstract

This study aimed to evaluate whether the single nucleotide polymorphisms of ATP-binding cassette subfamily G member 2 (*ABCG2*) and solute carrier family 2 member 9 (*SLC2A9*) affect individual blood uric acid levels using pyrosequencing. *ABCG2* (rs2231142, rs72552713, rs2231137), *SLC2A9* (rs3734553, rs3733591, rs16890979), and individual uric acid levels were prospectively analyzed in 250 healthy young Korean male participants. Prominent differences in uric acid levels of the alleles were observed in the *SLC2A9* rs3733591 polymorphism: wild-type (AA) vs. heterozygote (AG), 0.7 mg/dL (*p* < 0.0001); AA vs. mutant type (GG), 1.32 mg/dL (*p* < 0.0001); and AG vs. GG, 0.62 mg/dL (*p* < 0.01). In *ABCG2* single nucleotide polymorphisms (SNPs), the statistically significant differences in uric acid levels were only found in rs2231142 between CC vs. AA (1.06 mg/dL; *p* < 0.001), and CC vs. CA (0.59 mg/dL; *p* < 0.01). Serum uric acid levels based on the *ABCG2* and *SLC2A9* diplotype groups were also compared. The uric acid levels were the lowest in the CC/AA diplotype and highest in the AA/AG diplotype. In addition, the SNP *SLC2A9* rs3733591 tended to increase the uric acid levels when the *ABCG2* rs2231142 haplotypes were fixed. In conclusion, both the *ABCG2* rs2231142 and *SLC2A9* rs3733591 polymorphisms may additively elevate blood uric acid levels.

## 1. Introduction

Uric acid is an organic acid produced by endogenous purine metabolism, predominantly in the liver [1]. The main enzymes responsible for this process include phosphoribosyl pyrophosphate (PRPP) synthetase, purine nucleoside phosphorylase, xanthine oxidase (XO), and hypoxanthine-guanine phosphoribosyl transferase (HGPRT) [2]. Ribose-5-phosphate, synthesized from glycidic metabolism, is converted to PRPP by PRPP synthetase and is then converted to inosine monophosphate (IMP) [3,4]. IMP is converted to inosine and subsequently produces hypoxanthine and xanthine via XO. Uric acid is produced from xanthine by XO [5]. IMP can also get converted to adenosine monophosphate (AMP) and guanosine monophosphate (GMP). Xanthine can also be produced via the HGPRT and PRPP pathways, eventually producing uric acid [6,7,8].

During uric acid production, the reaction between superoxide and nitric oxide (NO) reduces the bioavailability of NO, which is accompanied by an increase in reactive oxygen species [9,10]. This may cause or deteriorate endothelial dysfunction, which may lead to cardiovascular disorders [10]. However, uric acid plays the major role of an antioxidant in human blood [11]; thus, a high uric acid concentration is related to the protective effect of neurodegenerative diseases, such as Alzheimer’s disease and Parkinson’s disease [12,13].

Uric acid is excreted mainly from the kidney (approximately 75%) by urate transporters located in the proximal tubules of the kidney [1]. In detail, most of the uric acid in the kidney is reabsorbed in the proximal tube (S1) after being filtered through the glomerulus. Fifty percent of the reabsorbed urate is secreted (S2) followed by 40% undergoing postsecretory reabsorption (S3) [1]. The role of transporters ABCG2, which is also known as breast cancer resistance protein (BCRP), and SLC2A9, which is also known as glucose transporter 9 (GLUT9), both of which are located in the tubular epithelial cells in the uric acid excretion, has been suggested [14,15,16,17] and several in vitro studies have identified the roles of SLC2A9 and ABCG2 transporters in uric acid disposition [18,19,20,21]. In BCRP knockout mice, uric acid levels were markedly higher than those in the wild type (AA) [17]. In an experiment involving kidney-specific *SLC2A9* knockout mice, uric acid excretion levels were more than seven times higher than those in the control [22]. The clinical functions of these transporters in uric acid levels have also been suggested in studies exploring the role of single nucleotide polymorphisms (SNPs) in *SLC2A9* (e.g., rs734553, rs3733591, and rs16890979) [23,24,25] and *ABCG2* (e.g., rs2231142, 72552713, and rs2231137) [23,26,27] and have been significantly linked with gout [28], implying the functional impacts of these polymorphisms on these transporters.

However, the combined effect of *SLC2A9* and *ABCG2* SNPs on human serum uric acid levels has not yet been confirmed. *SLC2A9* and *ABCG2* haplotypes are reported to play pivotal roles in the disposition of uric acid [26,28]. However, various factors, including sex, aging, acute and chronic diseases, diet, and medication, affect the levels of uric acid in human blood [29,30,31,32,33]. Furthermore, gout is predominantly found in the male population, and uric acid levels generally increase with aging [34,35,36,37]. Moreover, serum uric acid levels are known to be affected by certain medications (e.g., aspirin, levodopa, and hydrochlorothiazide), vigorous exercise, and foods containing a high amount of purine (e.g., meats, mushrooms, and dried peas). Therefore, this study aimed to evaluate the effects of genetic polymorphisms of *SLC2A9* and *ABCG2* on serum uric acid levels in healthy young male participants by controlling confounding factors.

## 2. Materials and Methods

### 2.1. Subjects and Study Design

Two hundred and fifty healthy young male participants between 20 and 35 years old were recruited. Female subjects were excluded to rule out the effects of hormones and sex on uric acid metabolism [38,39]. Only healthy young male participants were recruited, minimizing the effects of aging and chronic diseases on uric acid levels [38]. All subjects provided written informed consent before participation, and the study was approved by the Institutional Review Board of Anam Hospital, Korea University College of Medicine, Seoul, Korea (IRB no.: ED09100). Medical histories were determined, and physical examinations and routine laboratory tests were performed prior to selection. Subjects were excluded if they had a history or evidence of hepatic, renal, gastrointestinal, or hematologic abnormalities, any other acute or chronic disease, or an allergy to any drug. None of the subjects smoked tobacco or used continuous medication. No medications, herbal medicines, alcohol, grapefruit juice, or caffeinated beverages were permitted for 10 days before the study’s initiation and during the course of the study. Blood samples were obtained from all participants on day 0 (visit 1) and day 14 (visit 2), at around 8 AM, after 8 h or more of fasting. Serum uric acid levels were measured on those days, and the average value was obtained to minimize variations between days. All the samples were genotyped for both *SLC2A9* (rs734553, rs16890979, and rs3733591) and *ABCG2* (rs2231137, rs2231142, and rs72552713) polymorphisms using pyrosequencing.

### 2.2. DNA Extraction, Polymerase Chain Reaction, and Pyrosequencing

Genomic DNA was extracted from the blood samples using the GeneAll^®^ Exgene Blood SV kit (GeneAll Biotechnology, Inc., Seoul, Korea). Polymerase chain reaction (PCR) was performed using the below-mentioned protocol, and *ABCG2* and *SLC2A9* PCR primers used for pyrosequencing are described in Table A1. In brief, the PCR product mixture (a total of 30 μL) was composed of 3 μL of PCR buffer, 2 μL of dNTP (2.5 mM), 1 μL of forward and reverse primers (10 pmol) each, 5 units of Taq polymerase 0.3 μL (Intron Biotechnology, Inc., Seoul, Korea), and 50 ng genomic DNA, with the remaining volume containing distilled water. For pyrosequencing, 20 μL of the amplified PCR product was mixed with 5 μL of streptavidin beads, 40 μL of binding buffer, and 15 μL of distilled water in a single well in the 96-well PCR microplate, and incubated while shaking for 10 min using a micromixer (FINEPCR, Seoul, Korea) at room temperature. The beads containing the immobilized template were captured on the vacuum filter probes and subsequently transferred to each trough containing 70% ethanol, denaturation solution (0.2 M NaOH), washing solution (10 mM Tris-acetate, pH 7.6), and distilled water for 5 s. After draining the remaining liquid, the probe was placed onto the PyroMark Q96 HS plate (QIAGEN, Hilden, Germany) with a 40 μL annealing mixture containing 0.5 μL of 100 pmol sequencing primer. The PyroMark Q96 HS plate was heated at 85 °C for 2 min and cooled to 20 °C. The plate was then placed on a PyroMark Q96 MD pyrosequencer (QIAGEN) for sequence analysis [40]. The pyrosequencing accuracy was validated by direct DNA sequencing of randomly selected samples using the same genomic DNA.

### 2.3. Measurement of Uric Acid

Separate serum Vacutainer tubes (BD Vacutainers, Franklin Lakes, NJ, USA) were used to collect the blood samples from participants. Uric acid levels were measured using a Hitachi 7470 autoanalyzer (Hitachi, Tokyo, Japan) employing the uricase differential spectrophotometric method described in a previous study [41]. The average uric acid level during the two visits was used for the analysis.

### 2.4. Statistical Analysis

Data were expressed as mean value ± standard deviation (SD), and a *p* value < 0.05 was considered significant. Genetic equilibrium and linkage disequilibrium were tested according to the Hardy-Weinberg formula using SNPAlyze ver. 7.0 (DYNACOM Co., Ltd., Yokohama, Japan). In addition, haplotypes and diplotypes for *SLC2A9* and *ABCG2* polymorphisms were determined using this software. Statistical comparisons of uric acid levels among the *ABCG2* and/or *SLC2A9* genotypes were performed using the one-way analysis of variance test after a normality test (Shapiro–Wilk test; SAS ANOVA procedure), followed by Tukey’s post hoc analysis. The intraclass correlation coefficient (ICC) was calculated to assess the reliability and consistency of uric acid measurements between visits 1 and 2 [42]. Statistical analyses were performed using the statistical software package SAS, ver. 9.4 (SAS Institute, Cary, NC, USA).

## 3. Results

**Figure 1:**
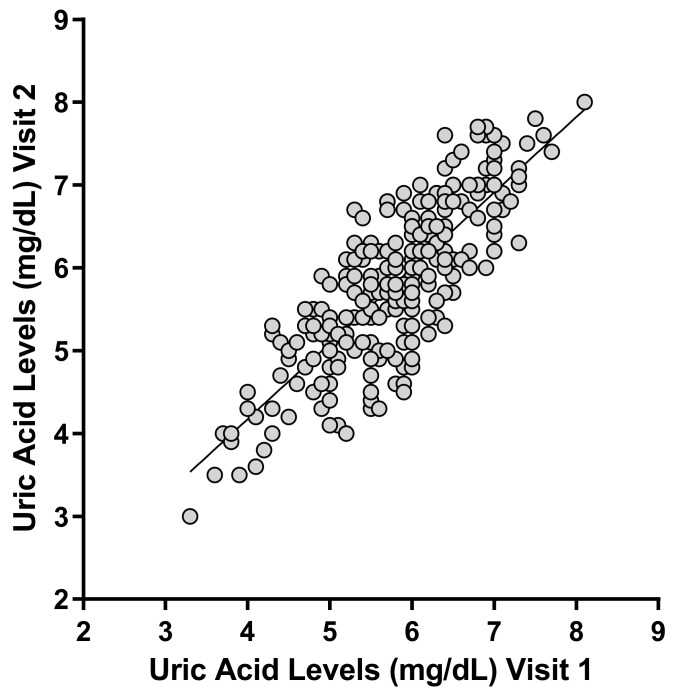
Observed uric acid levels in 250 healthy male subjects. The intraclass correlation coefficient between uric acid measured at visits 1 and 2 was 0.8075.

The demographic data of the participants, based on their *ABCG2* and *SLC2A9* genotypes, are described in Table 1. There were no significant differences in weight, height, age, or body mass index (BMI) among the genotypes of the analyzed SNPs. The measured serum uric acid levels did not differ significantly between visits 1 and 2 (ICC = 0.8075); therefore, the average value from each participant was used for subsequent analyses (Figure 1). The minor allele frequencies (MAF) of *ABCG2* rs2231137, rs2231142, and rs72552713 were 0.192, 0.258, and 0.018, respectively (Table 2). In addition, the MAF of *SLC2A9* rs734553, rs16890979, and rs3733591 were 0.020, 0.018, and 0.280, respectively (Table 2). None of the analyzed SNPs deviated from the Hardy-Weinberg equilibrium (Table 2).

**Figure 2:**
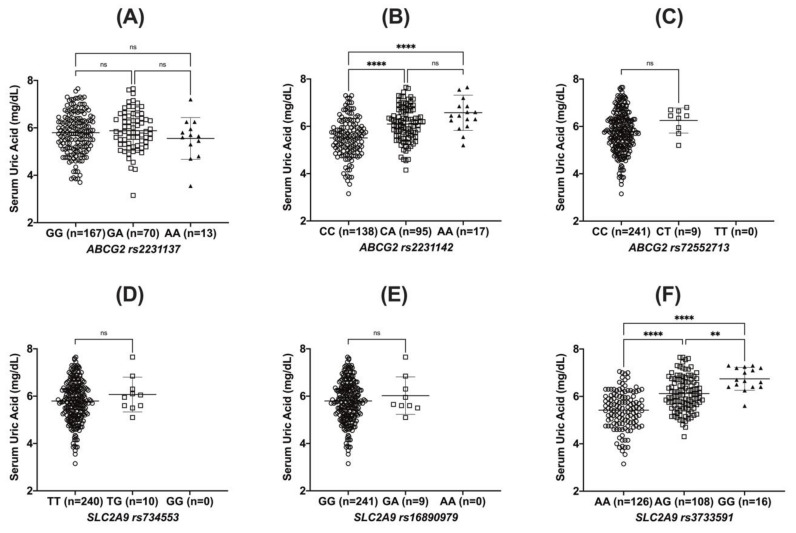
Comparisons between serum uric acid levels according to *ABCG2* (**A**–**C**) and *SLC2A9* (**D**–**F**) polymorphisms. The asterisks indicate the significant differences of groups (**, *p* < 0.01; ****, *p* < 0.0001, circle: homozygote wildtype, rectangle: heterozygote mutant, triangle: homozygote mutant).

When *ABCG2* polymorphisms were assessed to determine their association with serum uric acid levels, neither *ABCG2* rs2231137 nor rs72552713 influenced the variation in serum uric acid levels; however, *ABCG2* rs2231142 influenced it in a gene dose-dependent manner (5.51 mg/dL for CC, 6.10 mg/dL for CA, and 6.57 mg/dL for AA; *p* < 0.001) (Table 3 and Figure 2). Similar to *ABCG2* rs2231142, *SLC2A9* rs3733591 also influenced the serum uric acid levels in a gene dose-dependent manner (5.42 mg/dL for AA, 6.12 mg/dL for AG, and 6.74 mg/dL for GG; *p* < 0.001). However, neither *SLC2A9* rs734553 nor rs16890979 polymorphisms were found to be significantly associated with serum uric acid levels (Table 3 and Figure 2).

**Figure 3:**
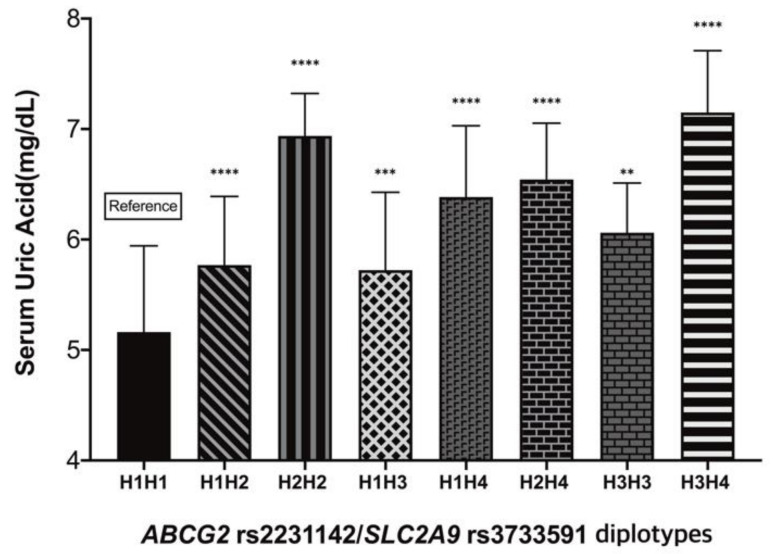
Comparisons between serum uric acid levels according to diplotype of *ABCG2* rs2231142 and *SLC2A9* rs3733591 polymorphisms. (**, *p* < 0.01; ***, *p* < 0.001; ****, *p* < 0.0001). The asterisks indicate the statistically different levels of uric acid in each diplotype when compared to the H1H1 diplotype (wild type).

As co-segregation behavior was observed in *ABCG2* rs2231142 and *SLC2A9* rs373359 polymorphisms on serum uric acid levels, the diplotypes of rs2231142 and rs373359 SNPs and their association with serum uric acid levels were also assessed. The four different haplotypes for both polymorphisms and their frequencies are listed in Table A2. The observed numbers and frequencies of diplotypes are shown in Table 4. In the diplotype distribution for *ABCG2* rs2231142 and *SLC2A9* rs3733591, the frequencies of the four diplotype groups were higher than 15% (29.6% for H1H1, 22.4% for H1H2, 17.2% for H1H3, and 17.6% for H1H4), and the frequencies of other observed diplotype groups (H2H2, H2H4, H3H3, and H3H4) were less than 4% in this population (Table 4). Serum uric acid levels were the lowest in the H1H1 group (5.16 ± 0.78 mg/dL) and highest in the H3H4 group (7.15 ± 0.56 mg/dL) (Table 4 and Figure 3).

## 4. Discussion

The results of this study revealed that both the *ABCG2* rs2231142 and *SLC2A9* rs3733591 polymorphisms were associated with variations in serum uric acid levels in this population. In addition, these two polymorphisms exhibited gene dose-dependent and additive effects on the elevation of serum uric acid levels. However, herein, other SNPs (*ABCG2* rs72552713, *ABCG2* rs2231137, *SLC2A9* rs734553, and *SLC2A9* rs16890979) did not affect serum uric acid levels.

Similarly, Tu et al. showed that the *SLC2A9* rs3733591 polymorphism significantly affected serum uric acid levels in Han Chinese and Solomon Islander populations [24]. They showed that the *SLC2A9* rs3733591 C allele is associated with the risk of gout and tophaceous gout in these populations. However, unlike our study, it failed to show a gene dose-dependent effect on serum uric acid levels: C-allele carriers showed higher serum uric acid levels than TT carriers (5.44 mg/dL), but the levels of serum uric acid in CC carriers (5.93 mg/dL) were comparable to those in CT carriers (5.91 mg/dL), suggesting a dominant inheritance model different from the current gene dose-dependent model. It has also been reported that the *ABCG2* rs2231142 polymorphism influences serum uric acid levels in a Han Taiwanese population [43]. Similarly, the results were focused only on serum uric acid levels following the dominant inheritance model (CC carriers vs. CA or AA carriers) and not the gene dose-dependent model. Although our results are similar to those of previous studies [24,43], the present study revealed obvious genetic effects of the *ABCG2* and *SLC2A9* polymorphisms on serum uric acid levels. These results may be evident because of the tailored study design used to adjust for confounding factors. Indeed, the literature reviewed indicated that serum uric acid is affected by various factors, including aging, sex, genetic, and environmental factors (e.g., BMI, blood pressure, fasting plasma glucose, red blood cell count, hemoglobin, white blood cell count, platelet count, and total cholesterol level) that may affect individual uric acid levels [44,45]. The results discussed herein were able to compare the individual uric acid levels after excluding confounding factors by enrolling only healthy young male participants, while previous studies could have been influenced by the abovementioned covariates.

In general, hyperuricemia is defined as serum uric acid levels exceeding 7.0 mg/dL, which is believed to increase the risk of gout and is related to metabolic syndromes (diabetes, hypertension, and obesity), chronic kidney disease, and various other cardiovascular diseases [46,47,48,49,50]. Interestingly, the results of our study showed that the average uric acid level in the AA/AG diplotype for *ABCG2* rs2231142/*SLC2A9* rs3733591 was 7.15 mg/dL. Considering that these values were measured in healthy young participants, we assumed that *ABCG2* rs2231142 and *SLC2A9* rs3733591 polymorphisms may have a clinical impact on uric acid levels. However, the average uric acid level in CC/AA (H1H1) diplotype for *ABCG2* rs2231142/*SLC2A9* rs3733591 was 5.16 mg/dL, which is within the normal range in the male population (3–6 mg/dL) [51]. In addition, it is plausible to assume that the *ABCG2* and *SLC2A9* transporters play a substantial role in normal serum uric acid levels.

Interestingly, SLC2A9 is an influx transporter that plays a role in the process of reabsorption of uric acid at the renal proximal tubule, thus influencing serum uric acid levels [26]. Considering the role of the transporter, its dysfunction by the polymorphism should cause a decrease in serum uric acid level, but *SLC2A9* rs3733591 polymorphism resulted in an increase in serum uric acid level in this study. We could not clearly explain these contradictory results. Additionally, we also could not find information to explain these findings during a literature review. *SLC2A9* genetic polymorphisms other than rs3733591 resulted in a decrease in gout susceptibility, but only the rs3733591 genetic polymorphism was related to increasing gout susceptibility in the opposite direction. In detail, there was no significant effect of the polymorphism on gout susceptibility in Easter Island and Western Polynesians, and Caucasians, including those from New Zealand, but it has a significant role in Korean, Solomon Island, and Maori populations [26].

The prevalence of *ABCG2* rs2231142 and *SLC2A9* rs3733591 was relatively higher (MAF ≥ 0.25) in the East Asian population (e.g., Korean, Japanese, and Chinese), compared to that of other ethnic groups [29,52,53,54,55,56,57]. However, the MAF of *ABCG2* rs2231142 was 0.11 for Caucasians and 0.02 for African Americans [53]. In addition, the MAF of *SLC2A9* rs3733591 was 0.16 for Caucasians and 0.12 for African Americans [43,56,57]. These results suggested that the two polymorphisms exhibit an ethnic difference in distribution and that their clinical impacts on uric acid levels may be more profound in East Asian populations. The impact of *ABCG2* rs2231142 and *SLC2A9* rs3733591 on uric acid levels based on ethnicity has not been confirmed yet. However, there is evidence that uric acid levels are relatively higher in Asians than those in other populations [58,59]. The reference interval for uric acid levels was the highest in Asians (3.9–9.1) and lowest in Hispanics (3.7–8.4) [58]. However, the risk of the prevalence of gout based on ethnicity is difficult to confirm from these results due to the many potential confounding factors [60,61,62].

Our study has some limitations that should be considered. Only healthy participants, not hyperuricemic or gout patients with high serum uric acid levels, were enrolled. In addition, sex and age are considered major risk factors influencing serum uric acid levels [43], but we enrolled only young male participants to exclude these confounding factors. However, these limitations originate from efforts to eliminate possible confounders.

In conclusion, the results of our study demonstrated that both the *ABCG2* rs2231142 and *SLC2A9* rs3733591 polymorphisms affected serum uric acid levels in a dose-dependent manner and elevated serum uric acid levels in healthy young male participants.

## Figures and Tables

**Table 1:** Demographics of the participants.

		Wild Type	Heterozygous Mutant	Homozygous Mutant	*p* Value
*ABCG2* rs2231137		GG (*n* = 167)	GA (*n* = 70)	AA (*n* = 13)	
	Weight (kg)	69.3 ± 6.7	69.4 ± 6.7	67.8 ± 6.0	0.714
	Height (cm)	175.4 ± 4.9	174.5 ± 4.9	174.7 ± 3.4	0.354
	Age (years)	24.5 ± 2.2	24.7 ± 2.4	24.6 ± 2.4	0.838
	BMI (kg/m^2^)	22.6 ± 2.4	22.8 ± 2.6	22.2 ± 2.3	0.609
*ABCG2* rs2231142		CC (*n* = 138)	CA (*n* = 95)	AA (*n* = 17)	
	Weight (kg)	68.5 ± 6.4	70.1 ± 6.5	70.7 ± 8.8	0.139
	Height (cm)	175.0 ± 4.6	175.2 ± 5.1	175.9 ± 4.8	0.728
	Age (years)	24.5 ± 2.3	24.7 ± 2.4	24.7 ± 1.8	0.724
	BMI (kg/m^2^)	22.4 ± 2.4	22.9 ± 2.5	22.9 ± 2.9	0.354
*ABCG2* rs72552713		CC (*n* = 241)	CT (*n* = 9)		
	Weight (kg)	69.2 ± 6.6	72.2 ± 8.6		0.174
	Height (cm)	175.0 ± 4.8	177.6 ± 3.3		0.121
	Age (years)	24.6 ± 2.3	24.4 ± 2.3		0.868
	BMI (kg/m^2^)	22.6 ± 2.4	22.9 ± 3.0		0.690
*SLC2A9* rs734553		TT (*n* = 240)	TG (*n* = 10)		
	Weight (kg)	69.2 ± 6.6	71.3 ± 7.9		0.323
	Height (cm)	175.1 ± 4.8	174.8 ± 5.3		0.831
	Age (years)	24.5 ± 2.3	25.4 ± 2.2		0.237
	BMI (kg/m^2^)	22.6 ± 2.4	23.4 ± 3.2		0.300
*SLC2A9* rs16890979		GG (*n* = 241)	GT (*n* = 9)		
	Weight (kg)	69.2 ± 6.6	70.0 ± 9.7		0.722
	Height (cm)	175.1 ± 4.8	174.8 ± 5.5		0.829
	Age (years)	24.5 ± 2.3	25.7 ± 2.1		0.139
	BMI (kg/m^2^)	22.6 ± 2.4	23.0 ± 3.9		0.605
*SLC2A9* rs3733591		AA (*n* = 126)	AG (*n* = 108)	GG (*n* = 16)	
	Weight (kg)	68.9 ± 6.1	70.1 ± 7.4	66.3 ± 5.1	0.071
	Height (cm)	174.8 ± 4.9	175.4 ± 4.8	175.6 ± 4.8	0.613
	Age (years)	24.5 ± 2.4	24.7 ± 2.3	24.1 ± 1.2	0.512
	BMI (kg/m^2^)	22.6 ± 2.4	22.8 ± 2.7	21.5 ± 1.6	0.147

**Table 2:** Genotypes of the participants and allele frequencies for the *ABCG2* and *SLC2A9* polymorphisms.

	Genotype Frequencies			Allele Frequencies		χ^2^	*p* Value
*ABCG2* rs2231137	GG	GA	AA	G	A		
*n*	167	70	13				
Frequencies	0.668	0.280	0.052	0.808	0.192	1.792	0.181
*ABCG2* rs2231142	CC	CA	AA	C	A		
*n*	138	95	17				
Frequencies	0.552	0.380	0.068	0.742	0.258	0.0022	0.963
*ABCG2* rs72552713	CC	CT	TT	C	T		
*n*	241	9	0				
Frequencies	0.964	0.036	0	0.982	0.018	2.248	0.134
*SLC2A9* rs734553	TT	TG	GG	T	G		
*n*	240	10	0				
Frequencies	0.96	0.04	0	0.980	0.020	1.666	0.197
*SLC2A9* rs16890979	GG	GA	AA	G	A		
*n*	241	9	0				
Frequencies	0.964	0.036	0	0.982	0.018	2.248	0.134
*SLC2A9* rs3733591	AA	AG	GG	A	G		
*n*	126	108	16				
Frequencies	0.504	0.432	0.064	0.720	0.280	0.946	0.331

χ^2^, comparison between observed numbers and expected numbers and the calculated Hardy-Weinberg equilibrium.

**Table 3:** Comparisons between serum uric acid levels according to the *ABCG2* and *SLC2A9* polymorphisms.

Genotypes	Alleles	*n*	Mean ± SD	95% CI
*ABCG2* rs2231137	GG	167	5.55 ± 0.88	(5.66–5.93)
	GA	70	5.88 ± 0.84	(5.68–6.08)
	AA	13	5.80 ± 0.88	(5.02–6.09)
	*p* value		0.448	
*ABCG2* rs2231142	CC	138	5.51 ± 0.83	(5.37–5.65)
	CA	95	6.10 ± 0.74	(5.94–6.25)
	AA	17	6.57 ± 0.74	(6.19–6.96)
	*p* value		< 0.001	
*ABCG2* rs72552713	CC	241	5.79 ± 0.87	(5.68–5.90)
	CT	9	6.25 ± 0.53	(5.84–6.65)
	*p* value		0.118	
*SLC2A9* rs734553	TT	240	5.80 ± 0.87	(5.69–5.91)
	TG	10	6.07 ± 0.74	(5.54–6.60)
	*p* value		0.328	
*SLC2A9* rs16890979	GG	241	5.80 ± 0.87	(5.69–5.91)
	GA	9	6.02 ± 0.79	(5.41–6.63)
	*p* value		0.449	
*SLC2A9* rs3733591	AA	126	5.42 ± 0.80	(5.28–5.56)
	AG	108	6.12 ± 0.75	(5.98–6.26)
	GG	16	6.74 ± 0.48	(6.48–7.00)
	*p* value		< 0.001	

CI, confidence interval.

**Table 4:** Frequencies and observed serum uric acid levels according to the diplotypes of *ABCG2* rs2231142 and *SLC2A9* rs3733591.

Diplotypes	Number	Frequency (%)	Mean ± SD	95% CI
H1H1	74	29.6	5.16 ± 0.78	5.00–5.32
H1H2	56	22.4	5.77 ± 0.62	5.62–5.92
H1H3	43	17.2	5.72 ± 0.70	5.53–5.92
H1H4	44	17.6	6.38 ± 0.65	6.21–6.56
H2H2	8	3.2	6.54 ± 0.51	6.66–7.22
H2H4	8	3.2	6.54 ± 0.51	6.18–6.91
H3H3	9	3.6	6.06 ± 0.45	5.76–6.36
H3H4	8	3.2	7.15 ± 0.56	6.75–7.56
H4H4	0	0	-	-

## Data Availability

The data presented in this study are available on request from the corresponding author. The data are not publicly available due to privacy and ethical reasons.

## Appendix A

**Table A1:** Description of the oligonucleotide primers used for PCR and pyrosequencing for the detection of *ABCG2* and *SLC2A9* SNPs.

	Primer	Sequences	Size, bp	PCR Tm, °C
*ABCG2* rs2231137	Forward	5′-GCTCATTGCCACACATTT-3′	188	60
	Reverse	B 5′-GAAGCCATTGGTGTTTCC-3′		
	Sequencing	5′-ATGTCGAAGTTTTTATCC-3′		
*ABCG2* rs2231142	Forward	B 5′-ATGTTGTGATGGGCACTCTGAC-3′	210	60
	Reverse	5′- TATCCACACAGGGAAAGTCCTACT-3′		
	Sequencing	5′-GAAGAGCTGCTGAGAACT-3′		
*ABCG2* rs72552713	Forward	5′-GTCTTAGCTGCAAGGAAAGAT-3′	166	60
	Reverse	B 5′-CCAAAGCACTTACCCATATAGA-3′		
	Sequencing	5′-AATGTAATTCAGGTTAYGTG-3′		
*SLC2A9* rs734553	Forward	B 5′-ACCCCATGATCTGATTATT-3′	176	60
	Reverse	5′-ACCCACCCTCATGATTTA-3′		
	Sequencing	5′-GGCTGACTGATTAGATCC-3′		
*SLC2A9* rs16890979	Forward	5′-GCATTAGACATGATGGACACTC-3′	223	60
	Reverse	B 5′-AGGCCATGGTGACAATCA-3′		
	Sequencing	5′-TTCTTGGGTAAAGCAGAC-3′		
*SLC2A9* rs3733591	Forward	5′-GCATTAGACATGATGGACACTC-3′	223	60
	Reverse	B 5′-AGGCCATGGTGACAATCA-3′		
	Sequencing	5′-GGAGGTCCTGGCTGAGAG-3′		

ABCG2, ATP-binding cassette subfamily G member 2; SLC2A9, solute carrier family 2 member 9; SNP, single nucleotide polymorphism; PCR, polymerase chain reaction; B, biotinylated at the end of the primer; Tm, melting temperature.

**Table A2:** Frequencies and number of haplotypes of *ABCG2* rs2231142 and *SLC2A9* rs3733591.

Haplotypes	*ABCG2* rs2231142	*SLC2A9* rs3733591	Frequencies
H1	C	A	0.5427
H2	C	G	0.1993
H3	A	A	0.1773
H4	A	G	0.0807

## References

[1] Fathallah-Shaykh S.A., Cramer M.T. (2014). Uric acid and the kidney. Pediatr. Nephrol..

[2] Cammalleri L., Malaguarnera M. (2007). Rasburicase represents a new tool for hyperuricemia in tumor lysis syndrome and in gout. Int. J. Med. Sci..

[3] Becker M.A., Puig J.G., Mateos F.A., Jimenez M.L., Kim M., Simmonds H. (1988). Inherited superactivity of phosphoribosylpyrophosphate synthetase: Association of uric acid overproduction and sensorineural deafness. Am. J. Med..

[4] Becker M.A., Losman M.J., Rosenberg A.L., Mehlman I., Levinson D.J., Holmes E.W. (1986). Phosphoribosylpyrophosphate synthetase superactivity: A study of five patients with catalytic defects in the enzyme. Arthritis Rheum..

[5] Wilcox W. (1996). Abnormal serum uric acid levels in children. J. Pediatr..

[6] Seegmiller J.E., Rosenbloom F.M., Kelley W.N. (1967). Enzyme Defect Associated with a Sex-Linked Human Neurological Disorder and Excessive Purine Synthesis. Science.

[7] Page T., Bakay B., Nissinen E., Nyhan W.L. (1981). Hypoxanthine--guanine phosphoribosyltransferase variants: Correlation of clinical phenotype with enzyme activity. J. Inherit. Metab. Dis..

[8] Sculley D.G., Dawson P.A., Emmerson B.T., Gordon R.B. (1992). A review of the molecular basis of hypoxanthine-guanine phosphoribosyltransferase (HPRT) deficiency. Hum. Genet..

[9] Sautin Y.Y., Johnson R.J. (2008). Uric Acid: The Oxidant-Antioxidant Paradox. Nucleosides Nucleotides Nucleic Acids.

[10] Kanellis J., Kang D.-H. (2005). Uric acid as a mediator of endothelial dysfunction, inflammation, and vascular disease. Semin. Nephrol..

[11] Ames B.N., Cathcart R., Schwiers E., Hochstein P. (1981). Uric acid provides an antioxidant defense in humans against oxidant- and radical-caused aging and cancer: A hypothesis. Proc. Natl. Acad. Sci. USA.

[12] Kim T.-S., Pae C.-U., Yoon S.-J., Jang W.-Y., Lee N.J., Kim J.-J., Lee S.-J., Lee C., Paik I.-H., Lee C.-U. (2006). Decreased plasma antioxidants in patients with Alzheimer’s disease. Int. J. Geriatr. Psychiatry.

[13] De Lau L.M.L., Koudstaal P.J., Hofman A., Breteler M.M. (2005). Serum uric acid levels and the risk of Parkinson disease. Ann. Neurol..

[14] Döring A., Gieger C., Mehta D., Gohlke H., Prokisch H., Coassin S., Fischer G., Henke K., Klopp N., Kronenberg F. (2008). SLC2A9 influences uric acid concentrations with pronounced sex-specific effects. Nat. Genet..

[15] Le M.T., Shafiu M., Mu W., Johnson R.J. (2008). SLC2A9--a fructose transporter identified as a novel uric acid transporter. Nephrol. Dial. Transplant..

[16] Itahana Y., Han R., Barbier S., Lei Z., Rozen S., Itahana K. (2014). The uric acid transporter SLC2A9 is a direct target gene of the tumor suppressor p53 contributing to antioxidant defense. Oncogene.

[17] Hosomi A., Nakanishi T., Fujita T., Tamai I. (2012). Extra-Renal Elimination of Uric Acid via Intestinal Efflux Transporter BCRP/ABCG2. PLoS ONE.

[18] Enomoto A., Kimura H., Chairoungdua A., Shigeta Y., Jutabha P., Cha S.H., Hosoyamada M., Takeda M., Sekine T., Igarashi T. (2002). Molecular identification of a renal urate–anion exchanger that regulates blood urate levels. Nature.

[19] Anzai N., Ichida K., Jutabha P., Kimura T., Babu E., Jin C.J., Srivastava S., Kitamura K., Hisatome I., Endou H. (2008). Plasma Urate Level Is Directly Regulated by a Voltage-driven Urate Efflux Transporter URATv1 (SLC2A9) in Humans. J. Biol. Chem..

[20] Kis E., Nagy T., Jani M., Molnar E., Janossy J., Ujhellyi O., Nemet K., Heredi-Szabo K., Krajcsi P. (2008). Leflunomide and its metabolite A771726 are high affinity substrates of BCRP: Implications for drug resistance. Ann. Rheum. Dis..

[21] Otero J.A., Miguel V., González-Lobato L., Villalba R.G., Espín J.C., Prieto J.G., Merino G., Álvarez A.I. (2016). Effect of bovine ABCG2 polymorphism Y581S SNP on secretion into milk of enterolactone, riboflavin and uric acid. Animal.

[22] Auberson M., Stadelmann S., Stoudmann C., Seuwen K., Koesters R., Thorens B., Bonny O. (2018). SLC2A9 (GLUT9) mediates urate reabsorption in the mouse kidney. Pflugers. Arch..

[23] Kolz M., Johnson T., Sanna S., Teumer A., Vitart V., Perola M., Mangino M., Albrecht E., Wallace C., Farrall M. (2009). Meta-Analysis of 28,141 Individuals Identifies Common Variants within Five New Loci That Influence Uric Acid Concentrations. PLoS Genet..

[24] Tu H.-P., Chen C.-J., Tovosia S., Ko A.M.-S., Lee C.-H., Ou T.-T., Lin G.-T., Chang S.-J., Chiang S.-L., Chiang H.-C. (2010). Associations of a non-synonymous variant in SLC2A9 with gouty arthritis and uric acid levels in Han Chinese subjects and Solomon Islanders. Ann. Rheum. Dis..

[25] Meng Q., Yue J., Shang M., Shan Q., Qi J., Mao Z., Li J., Zhang F., Wang B., Zhao T. (2015). Correlation of GLUT9 Polymorphisms with Gout Risk. Medicine.

[26] Lukkunaprasit T., Rattanasiri S., Turongkaravee S., Suvannang N., Ingsathit A., Attia J., Thakkinstian A. (2020). The association between genetic polymorphisms in ABCG2 and SLC2A9 and urate: An updated systematic review and meta-analysis. BMC Med. Genet..

[27] Tu H.-P., Ko A.M.-S., Chiang S.-L., Lee S.-S., Lai H.-M., Chung C.-M., Huang C.-M., Lee C.-H., Kuo T.-M., Hsieh M.-J. (2014). Joint Effects of Alcohol Consumption and *ABCG2* Q141K on Chronic Tophaceous Gout Risk. J. Rheumatol..

[28] Takei R., Cadzow M., Markie D., Bixley M., Phipps-Green A., Major T.J., Li C., Choi H.K., Li Z., Hu H. (2021). Trans-ancestral dissection of urate- and gout-associated major loci SLC2A9 and ABCG2 reveals primate-specific regulatory effects. J. Hum. Genet..

[29] Liu J., Yang W., Li Y., Wei Z., Dan X. (2020). ABCG2 rs2231142 variant in hyperuricemia is modified by SLC2A9 and SLC22A12 polymorphisms and cardiovascular risk factors in an elderly community-dwelling population. BMC Med. Genet..

[30] Lee J.J., Ahn J., Hwang J., Han S.W., Lee K.N., Kim J.B., Lee S., Na J.O., Lim H.E., Kim J.W. (2015). Relationship between uric acid and blood pressure in different age groups. Clin. Hypertens..

[31] Zand S., Shafiee A., Boroumand M., Jalali A., Nozari Y. (2013). Serum Uric Acid Is Not an Independent Risk Factor for Premature Coronary Artery Disease. Cardiorenal Med..

[32] Choi J.W.J., Ford E.S., Gao X., Choi H.K. (2007). Sugar-sweetened soft drinks, diet soft drinks, and serum uric acid level: The third national health and nutrition examination survey. Arthritis Rheum..

[33] Reyes A.J. (2005). The increase in serum uric acid concentration caused by diuretics might be beneficial in heart failure. Eur. J. Heart Fail..

[34] Ene-Stroescu D., Gorbien M.J. (2005). Gouty arthritis. A primer on late-onset gout. Geriatrics.

[35] Mikuls T.R., Farrar J.T., Bilker W.B., Fernandes S., Schumacher H.R., Saag K.G. (2005). Gout epidemiology: Results from the UK General Practice Research Database, 1990-1999. Ann. Rheum. Dis..

[36] Lawrence R.C., Helmick C.G., Arnett F.C., Deyo R.A., Felson D.T., Giannini E.H., Heyse S.P., Hirsch R., Hochberg M.C., Hunder G.G. (1998). Estimates of the prevalence of arthritis and selected musculoskeletal disorders in the United States. Arthritis Rheum..

[37] Wallace K.L., Riedel A.A., Joseph-Ridge N., Wortmann R. (2004). Increasing prevalence of gout and hyperuricemia over 10 years among older adults in a managed care population. J. Rheumatol..

[38] Lin X., Wang X., Li X., Song L., Meng Z., Yang Q., Zhang W., Gao Y., Yang Z., Cai H. (2019). Gender- and Age-Specific Differences in the Association of Hyperuricemia and Hypertension: A Cross-Sectional Study. Int. J. Endocrinol..

[39] Sacks D., Baxter B., Campbell B.C.V., Carpenter J.S., Cognard C., Dippel D., Eesa M., Fischer U., Hausegger K., Hirsch J.A. (2018). Multisociety Consensus Quality Improvement Revised Consensus Statement for Endovascular Therapy of Acute Ischemic Stroke. Int. J. Stroke.

[40] Kim K.-A., Joo H.-J., Park J.-Y. (2010). ABCG2 polymorphisms, 34G>A and 421C>A in a Korean population: Analysis and a comprehensive comparison with other populations. J. Clin. Pharm. Ther..

[41] Kim K.-A., Joo H.-J., Park J.-Y. (2010). Effect of ABCG2 genotypes on the pharmacokinetics of A771726, an active metabolite of prodrug leflunomide, and association of A771726 exposure with serum uric acid level. Eur. J. Clin. Pharmacol..

[42] Kim K.-A., Song W.-G., Lee H.-M., Joo H.-J., Park J.-Y. (2013). Effect of P2Y1 and P2Y12 genetic polymorphisms on the ADP-induced platelet aggregation in a Korean population. Thromb. Res..

[43] Cheng S.-T., Wu S., Su C.-W., Teng M.-S., Hsu L.-A., Ko Y.-L. (2017). Association of ABCG2 rs2231142-A allele and serum uric acid levels in male and obese individuals in a Han Taiwanese population. J. Formos. Med. Assoc..

[44] Hall A.P., Barry P.E., Dawber T.R., McNamara P.M. (1967). Epidemiology of gout and hyperuricemia. A long-term population study. Am. J. Med..

[45] Campion E.W., Glynn R.J., DeLabry L.O. (1987). Asymptomatic hyperuricemia. Risks and consequences in the normative aging study. Am. J. Med..

[46] Zhang S., Wang Y., Cheng J., Huangfu N., Zhao R., Xu Z., Zhang F., Zheng W., Zhang D. (2019). Hyperuricemia and Cardiovascular Disease. Curr. Pharm. Des..

[47] Yip K., Cohen R.E., Pillinger M.H. (2020). Asymptomatic hyperuricemia: Is it really asymptomatic?. Curr. Opin. Rheumatol..

[48] Álvarez-Lario B., Alonso-Valdivielso J.L. (2014). Hyperuricemia and gout; the role of diet. Nutr. Hosp..

[49] Petreski T., Ekart R., Hojs R., Bevc S. (2020). Hyperuricemia, the heart, and the kidneys—To treat or not to treat?. Ren. Fail..

[50] Li C., Hsieh M.-C., Chang S.-J. (2013). Metabolic syndrome, diabetes, and hyperuricemia. Curr. Opin. Rheumatol..

[51] De Becker B., Borghi C., Burnier M., van de Borne P. (2019). Uric acid and hypertension: A focused review and practical recom-mendations. J. Hypertens..

[52] Yang H.J., Liu M., Kim M.J., Park S. (2021). The haplotype of *SLC2A9*_rs3733591, *PKD2*_rs2725220 and *ABCG2*_rs2231142 increases the hyperuricaemia risk and alcohol, chicken and processed meat intakes and smoking interact with its risk. Int. J. Food Sci. Nutr..

[53] Kobayashi D., Ieiri I., Hirota T., Takane H., Maegawa S., Kigawa J., Suzuki H., Nanba E., Oshimura M., Terakawa N. (2004). Functional assessment of ABCG2 (BCRP) gene polymorphisms to protein expression in human placenta. Drug Metab. Dispos..

[54] Yang Y., Jia J., Sun Z., Liu C., Li Z., Xiao Y., Yu J., Du F., Shi Y., Sun J. (2021). Polymorphism of FGD4 and myelosuppression in patients with esophageal squamous cell carcinoma. Futur. Oncol..

[55] Urano W., Taniguchi A., Anzai N., Inoue E., Sekita C., Endou H., Kamatani N., Yamanaka H. (2010). Association between GLUT9 and gout in Japanese men. Ann. Rheum. Dis..

[56] Hollis-Moffatt J.E., Gow P.J., Harrison A.A., Highton J., Jones P.B., Stamp L.K., Dalbeth N., Merriman T.R. (2011). The SLC2A9 nonsynonymous Arg265His variant and gout: Evidence for a population-specific effect on severity. Arthritis Res. Ther..

[57] McArdle P.F., Parsa A., Chang Y.-P.C., Weir M.R., O’Connell J.R., Mitchell B., Shuldiner A. (2008). Association of a common nonsynonymous variant in GLUT9 with serum uric acid levels in old order amish. Arthritis Rheum..

[58] Lim E., Miyamura J., Chen J.J. (2015). Racial/Ethnic-Specific Reference Intervals for Common Laboratory Tests: A Comparison among Asians, Blacks, Hispanics, and White. Hawaii J. Med. Public Health.

[59] Singh J.A. (2013). Racial and Gender Disparities Among Patients with Gout. Curr. Rheumatol. Rep..

[60] Wang H., Wang L., Xie R., Dai W., Gao C., Shen P., Huang X., Zhang F., Yang X., Ji G. (2014). Association of Serum Uric Acid with Body Mass Index: A Cross-Sectional Study from Jiangsu Province, China. Iran. J. Public Health.

[61] Palmer T.M., Nordestgaard B.G., Benn M., Tybjaerg-Hansen A., Smith G.D., Lawlor D.A., Timpson N.J. (2013). Association of plasma uric acid with ischaemic heart disease and blood pressure: Mendelian randomisation analysis of two large cohorts. Bmj.

[62] Parsa A., Brown E., Weir M.R., Fink J.C., Shuldiner A.R., Mitchell B.D., McArdle P.F. (2012). Genotype-based changes in serum uric acid affect blood pressure. Kidney Int..

